# Right and Left Ventricular Strain Patterns After the Atrial Switch Operation for D-Transposition of the Great Arteries—A Magnetic Resonance Feature Tracking Study

**DOI:** 10.3389/fcvm.2019.00039

**Published:** 2019-04-09

**Authors:** Barbara Elisabeth Ursula Burkhardt, Christian Johannes Kellenberger, Francesca Daniela Franzoso, Julia Geiger, Angela Oxenius, Emanuela Regina Valsangiacomo Buechel

**Affiliations:** ^1^Department of Surgery, Pediatric Cardiology, Pediatric Heart Center, University Children's Hospital Zurich, Zurich, Switzerland; ^2^Children's Research Center, University Children's Hospital Zurich, Zurich, Switzerland; ^3^Department of Diagnostic Imaging, University Children's Hospital Zurich, Zurich, Switzerland

**Keywords:** transposition of great arteries (TGA), atrial switch operation, cardiovascular magnetic resonance (CMR), strain, systemic right ventricle

## Abstract

**Introduction:** Adult survivors of the atrial switch operation for transposition of the great arteries present with a systemic morphologic right ventricle and a subpulmonary morphologic left ventricle. This physiology can be considered a model for the effects of long-term right ventricular pressure overload and of decreased left ventricular afterload. We aimed to determine the impact of these chronically altered loading conditions on myocardial deformation of the ventricles.

**Materials and methods:** Two-dimensional steady state free precession cine images of 29 patients after atrial repair (age 29 ± 7 years) and 19 controls (24 ± 10 years; n.s.) were post-processed with feature tracking software (TomTec 2D CPA). Volumes, ejection fractions, global and free wall longitudinal and circumferential strains of both ventricles were compared between both groups.

**Results:** Systemic right ventricular global longitudinal strain was decreased in patients compared to controls (−12.9 ± 3.3% vs. −18.9 ± 4.6%, *p* < 0.001), while right ventricular circumferential strain was unchanged (−15.8 ± 3.4% vs. −15.1 ± 5%; n.s.). Left ventricular longitudinal strain was similar in both groups (−17 ± 5.6% vs. −17.5 ± 4.6%; n.s.), but global left ventricular circumferential strain was lower in patients (−20.7 ± 4.1% vs. −27.3 ± 4.5%, *p* < 0.001). The systemic right ventricle, compared to the systemic left ventricle, showed decreased global longitudinal (*p* < 0.001) and circumferential strain (*p* < 0.001). The subpulmonary left ventricle, compared to the subpulmonary right ventricle, demonstrated similar longitudinal (*p* = 0.223) but higher circumferential strain (*p* < 0.001).

**Conclusions:** In patients after atrial switch repair for transposition of the great arteries, the systemic right ventricle shows poor longitudinal strain, but maintains normal right ventricular circumferential strain. The left ventricle shows higher circumferential strain than the right ventricle, in both systemic and subpulmonary positions.

## Introduction

The atrial switch operation according to Senning or Mustard has been the surgical repair technique of choice for transposition of the great arteries (TGA) for many years (1). In the 1980's, it was replaced by the arterial switch operation (2). The Senning procedure results in a subaortic right ventricle (RV) pumping into the systemic circulation and a subpulmonary left ventricle (LV). During follow-up, the chronic pressure overload may cause deterioration of RV function with impaired clinical functional status and eventually RV failure and increased mortality (3). LV function can be compromised by RV dysfunction due to negative ventriculo-ventricular interaction (4). Therefore, functional assessment of both ventricles is of high clinical importance in this population (5).

Cardiac magnetic resonance (CMR) has evolved to be the reference method for calculation of RV volumes and function, due to its high accuracy and reproducibility (6).

Magnetic resonance feature tracking (MRFT) enables quantification of the segmental and global motion of the myocardium from CMR cine images (7). MRFT is similar to echocardiographic speckle tracking analysis, which has been demonstrated to be a useful diagnostic and prognostic modality in many different congenital heart lesions, including those with a systemic RV (8–11). However, reports of MRFT in patients with systemic right ventricles are still limited (12, 13).

The aim of this study was to examine regional deformation of the ventricles in patients after atrial repair (Senning) and to compare it with that of individuals with normal cardiac anatomy (controls). We hypothesized that the RV and LV in Senning patients show different deformation patterns than normal ventricles due to their reversed pressure loads.

## Materials and Methods

### Patient Population

All consecutive patients with TGA after atrial switch operation undergoing a CMR examination between June, 2009 and March, 2016 were considered for inclusion in the study. Exclusion criteria consisted of evidence of chronic tachyarrhythmias or arrhythmias at the time of image acquisition, significantly impaired function with an ejection fraction (EF%) of either ventricle of <30%, significant baffle leak with a pulmonary to systemic blood flow ratio > 1.3: 1, significant baffle stenosis with evident venous dilation and/or reversed flow in the azygous vein, subpulmonary LV outflow tract obstruction. Baseline characteristics, CMR data including ventricular volumes, EF%, flow volumes, and strains in longitudinal and circumferential direction, as well as follow up cardiopulmonary exercise (CPEX) data were collected / measured.

Healthy volunteers and patients in whom cardiac pathology was ruled out by CMR, such as patients who were imaged for suspected vascular rings, were recruited as normal controls.

### Image Acquisition

All subjects underwent CMR on a 1.5 Tesla system (Signa MR/i Twinspeed or Discovery MR 450, GE Healthcare, Milwaukee, WI, USA) using a 32-channel phased-array cardiac coil and vector cardiogram for retrospective cardiac gating.

Cine Steady State Free Precession (SSFP) images were acquired in a horizontal long-axis plane showing both ventricles and both atria as well as in a stack of 12–13 adjacent short-axis slices through both ventricles from the cardiac base to the apex, with a slice thickness of 8 mm and gap of 0–2 mm. All images were acquired in end-expiratory breath-holding.

The parameters of the SSFP sequence were as follows: 40 reconstructed phases/cardiac cycle, TE 1.5–1.8 ms, TR 2.8–3.1 ms, flip angle 45°, bandwidth 125 kHz, matrix 224 × 224, number of excitations 1, field of view 250–350 mm, views per segment 6–12 depending on heart rate. Parameters were optimized for obtaining a temporal resolution of <30 ms (mean 26.7 ms). Cine phase contrast images were obtained in through planes perpendicular to the ascending aorta and main pulmonary artery, in breath-holding technique, for internal validation of stroke volumes.

### Image Analysis

Ventricular volumes and ejection fractions were calculated offline on a separate workstation with a dedicated software (QMass, Medis Suite 2.0.16.0, MEDIS, Medical Imaging Systems, Leiden, The Netherlands). The endsystolic and enddiastolic phases were identified visually in a midventricular short axis slice, the endocardial contours were manually traced; volumes and ejection fractions were calculated as previously described and indexed to body surface area (14). On phase contrast images, vessel contours were traced in all phases, and flow volumes were calculated (QFlow, Medis Suite 2.0.16.0, MEDIS, Medical Imaging Systems, Leiden, The Netherlands) to determine residual shunts.

**Figure 1:**
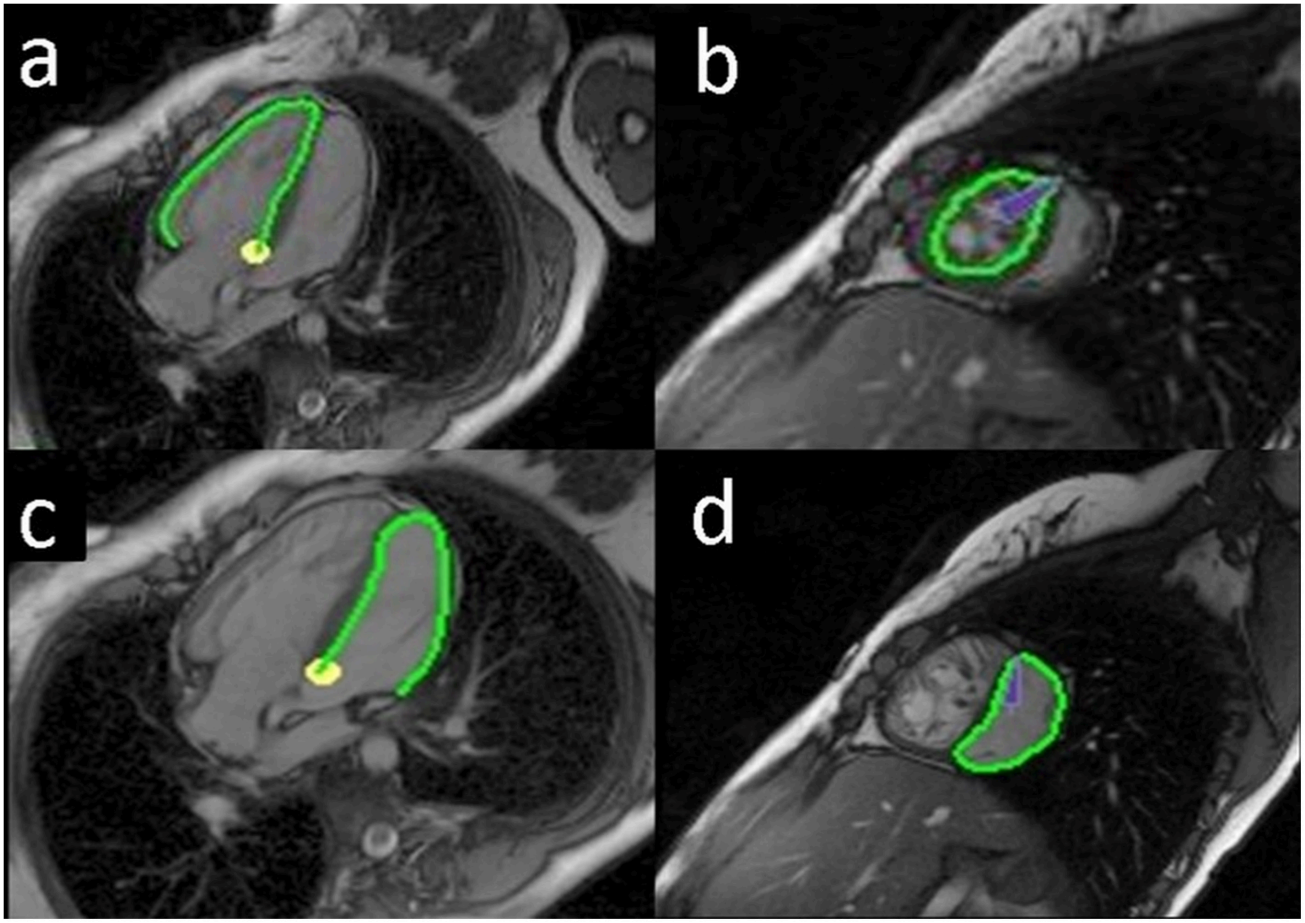
Endocardial tracing of the ventricles in a Senning patient. The green line shows the endocardial border tracing of the systemic right ventricle (RV), horizontal long axis **(a)**; systemic RV, short axis **(b)**; subpulmonary left ventricle (LV), horizontal long axis **(c)**; subpulmonary LV, short axis **(d)**.

Feature tracking analysis was performed using a dedicated software (TomTec 2D Cardiac Performance Analysis MR 1.0.1, TomTec, Unterschleissheim, Germany). Endocardial contours of both ventricles were traced manually on an end-diastolic image frame (Figure 1), and subsequently semi-automatic processing provided tracked endocardial borders throughout the cardiac cycle. If automatic tracking was inadequate, endocardial borders were corrected manually and automatic tracking restarted until the tracked borders fitted all cardiac phases.

Longitudinal strain analysis was performed on the horizontal long-axis view (four chamber view) of both ventricles. For the LV, the endocardial border was traced beginning at the septal mitral annulus, along the apex, and ending at the lateral mitral ring in a clockwise direction. For the RV, a mirror image was created in order to similarly trace from the septal tricuspid annulus, along the apex, and ending at the lateral tricuspid ring in a clockwise direction.

Circumferential strain was measured for both ventricles on one midventricular slice on short axis images. Midventricular location was chosen to minimize the out-of-plane motion of the region of interest (15).

The software provided values of global longitudinal and/or circumferential strain for the LV and the RV by averaging all segmental values. Regional segmental values were provided for the LV following the American Heart Association 17-segment model. We used a “mirror image” nomenclature for defining the segments of the RV (16). Systemic ventricle twist was calculated as the difference of apical and basal systolic rotations, and torsion was calculated by dividing twist by the distance between the respective image planes (17).

Image quality was assessed for each slice and classified as: 2 = good, 1 = moderate, 0 = inadequate. Images of inadequate quality were excluded from analysis.

Intraobserver variability was tested by one investigator (BB) measuring twice, on different days at least 8 weeks apart. Interobserver variability was assessed from measurements of two independent investigators (BB, EV) blinded to each other's results.

### Statistics

Continuous data are expressed as mean and standard deviation (SD). The one-sample Kolmogorov-Smirnoff test was used to test for normal distribution. The Student's *t*-test was used to compare data between groups. For parameters that were not normally distributed, the Mann-Whitney *U*-test was used for comparison statistics. Correlations were calculated using Pearson's correlation coefficient. Intra- and interobserver variability was examined with Bland-Altman analyses (18) and intraclass correlation coefficients (type C, two-way mixed effects model, average measures). Coefficients of variation (CV) were calculated as the standard deviations of differences between two measurements, divided by the respective means of two measurements. Statistical significance was defined as a two-sided *p*-value < 0.05. SPSS software, versions 22 and 24 (IBM Corporation, Armonk, NY, USA), was used for statistical analyses.

## Results

### Patient Characteristics

Twenty-nine patients after the atrial switch operation (Senning group) and 19 healthy normal controls (control group) were recruited in the study. Subject characteristics did not differ in terms of age, sex, height, weight, or heart rate (Table 1). In 4 patients, associated cardiac anomalies were addressed during the Senning operation, including closure of ventricular septal defects in 2, closure of ventricular septal defects and pulmonary artery de-banding in1, and resection of subvalvular pulmonary stenosis in one. Three patients had had cardiac interventions after the Senning procedure, consisting of radiofrequency ablation in 2, atrial septal occluder implantation for baffle leak in one. None underwent repeated cardiac surgery. Cardiac medication at the time of CMR consisted of ACE inhibitors in 7 patients and an angiotensin receptor blocker in one.

**Table 1:** Subject characteristics.

	**Senning; *n* = 29**	**Control; *n* = 19**	***p***
Age [years]	29 ± 7	24 ± 10	0.05
Male [number (%)]	17 (59%)	10 (53%)	0.46
Height [cm]	167.9 ± 10.2	172.4 ± 13.9	0.20
Weight [kg]	68 ± 13.5	64.6 ± 12.7	0.39
Heart rate [bpm]	64 ± 14	70 ± 16	0.12

*Values are expressed as mean ± standard deviation*.

### Ventricular Volumes and Ejection Fractions

Senning patients presented larger RV end-diastolic volumes (RV EDV) (*p* < 0.001) and lower RV EF% (*p* < 0.001) than controls (Table 2).

**Table 2:** Ventricular volumes and ejection fractions for the right and the left ventricle in their anatomic positions (upper half) and in their functional positions (lower half).

	**Senning; *n* = 29**	**Control; *n* = 19**	***p***
RV EDVI [ml/m^2^]	114 ± 24	84 ± 16	**<0.001**
RV ESVI [ml/m^2^]	68 ± 18	40 ± 9	**0.015**
RV EF [%]	40 ± 5	53 ± 6	**<0.001**
LV EDVI [ml/m^2^]	75 ± 15	88 ± 16	**<0.01**
LV ESVI [ml/m^2^]	30 ± 8	37 ± 9	0.514
LV EF [%]	60 ± 6	58 ± 5	0.159
RV/LV EDVR	1.53 ± 0.21	0.96 ± 0.10	**0.001**
SV EDVI [ml/m^2^]	114 ± 24	88 ± 16	**<0.001**
SV ESVI [ml/m^2^]	68 ± 18	37 ± 9	**0.022**
SV EF [%]	40 ± 5	58 ± 5	**<0.001**
SPV EDVI [ml/m^2^]	75 ± 15	84 ± 16	**<0.05**
SPV ESVI [ml/m^2^]	30 ± 8	40 ± 9	0.831
SPV EF [%]	60 ± 6	53 ± 6	**<0.001**

*Values are expressed as mean ± standard deviation. EDVI, end-diastolic volume index; EDVR, end-diastolic volume ratio; EF, ejection fraction; ESVI, end-systolic volume index; LV, left ventricle; RV, right ventricle; SPV, subpulmonary ventricle; SV, systemic ventricle. p values in bold type denote statistically significant differences*.

LV volumes were smaller in end-diastole (LV EDV) in the Senning group than in the control group (*p* < 0.01). No difference was observed for LV EF% (*p* = 0.43).

Comparing the ventricles in their functional positions, RV in systemic position were larger than the normal systemic LV (*p* < 0.001). The systemic RV had lower EF% than the systemic LV (*p* < 0.001).

The subpulmonary LV in the Senning group were smaller than the subpulmonary RV in the control group (*p* < 0.05) and showed higher EF% (*p* = 0.001).

### Longitudinal Annular Plane Displacement

Tricuspid annular plane systolic excursion (APSE) was lower in the Senning than in the control group (8 ± 2.5 vs. 16.2 ± 3.3 mm; *p* < 0.001), while mitral APSE was not significantly different (11.6 ± 3.5 vs. 10.1 ± 2.7 mm; *p* = 0.126). Senning patients showed lower APSE of their systemic (8 ± 2.5 vs. 10.1 ± 2.7 mm; *p* < 0.001) and also lower APSE of their subpulmonary ventricles (11.6 ± 3.5 vs. 16.2 ± 3.3 mm; *p* < 0.001).

### Strain Measurements

**Figure 2:**
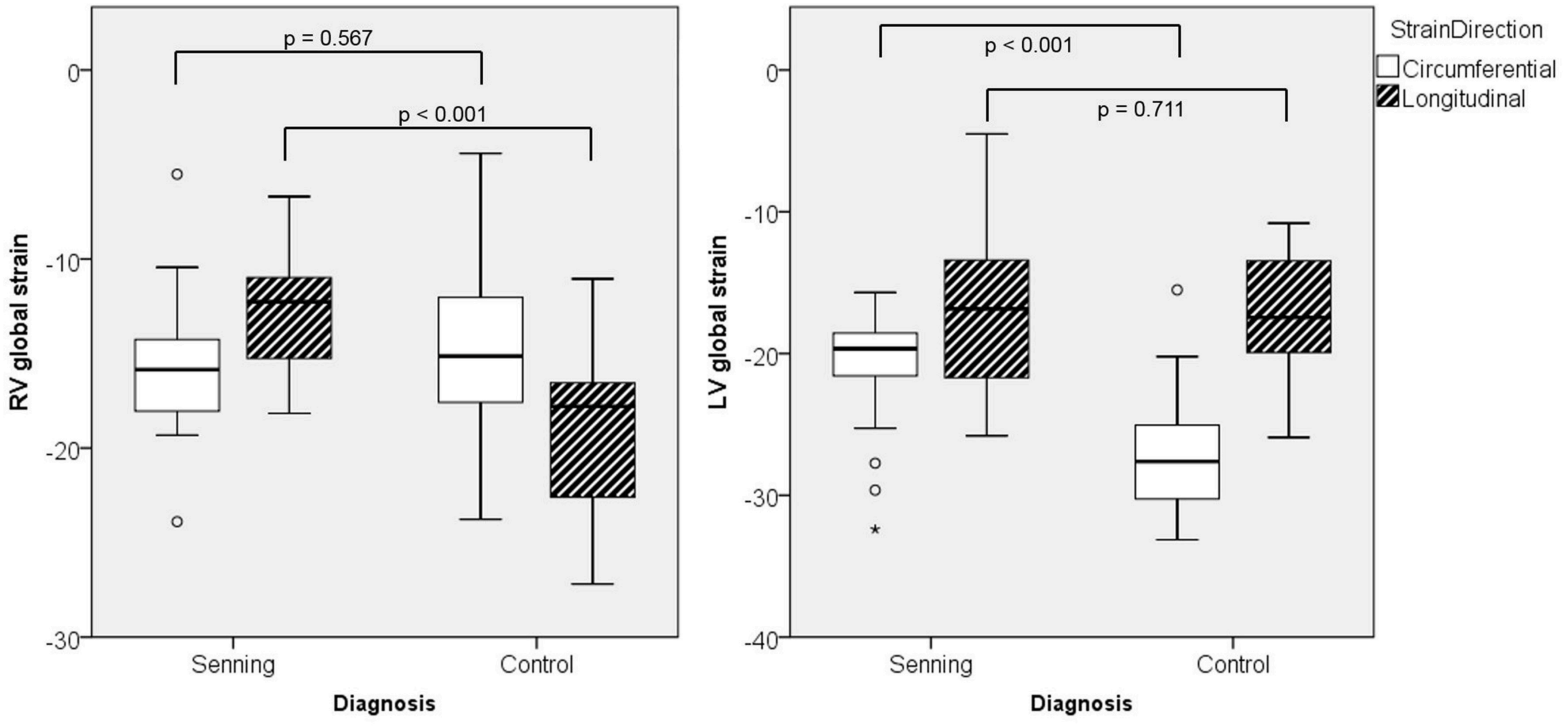
Maximal global strain for right (RV) and left ventricles (LV) in Senning patients and controls.

The systemic RV (Senning) showed lower global longitudinal strain (*p* < 0.001) compared to normal RV. No difference was found for RV global circumferential strain (Figure 2; Supplementary Table 1).

The subpulmonary LV (Senning) had similar global longitudinal strains but lower global circumferential strains than the normal systemic LV (*p* < 0.001).

**Figure 3:**
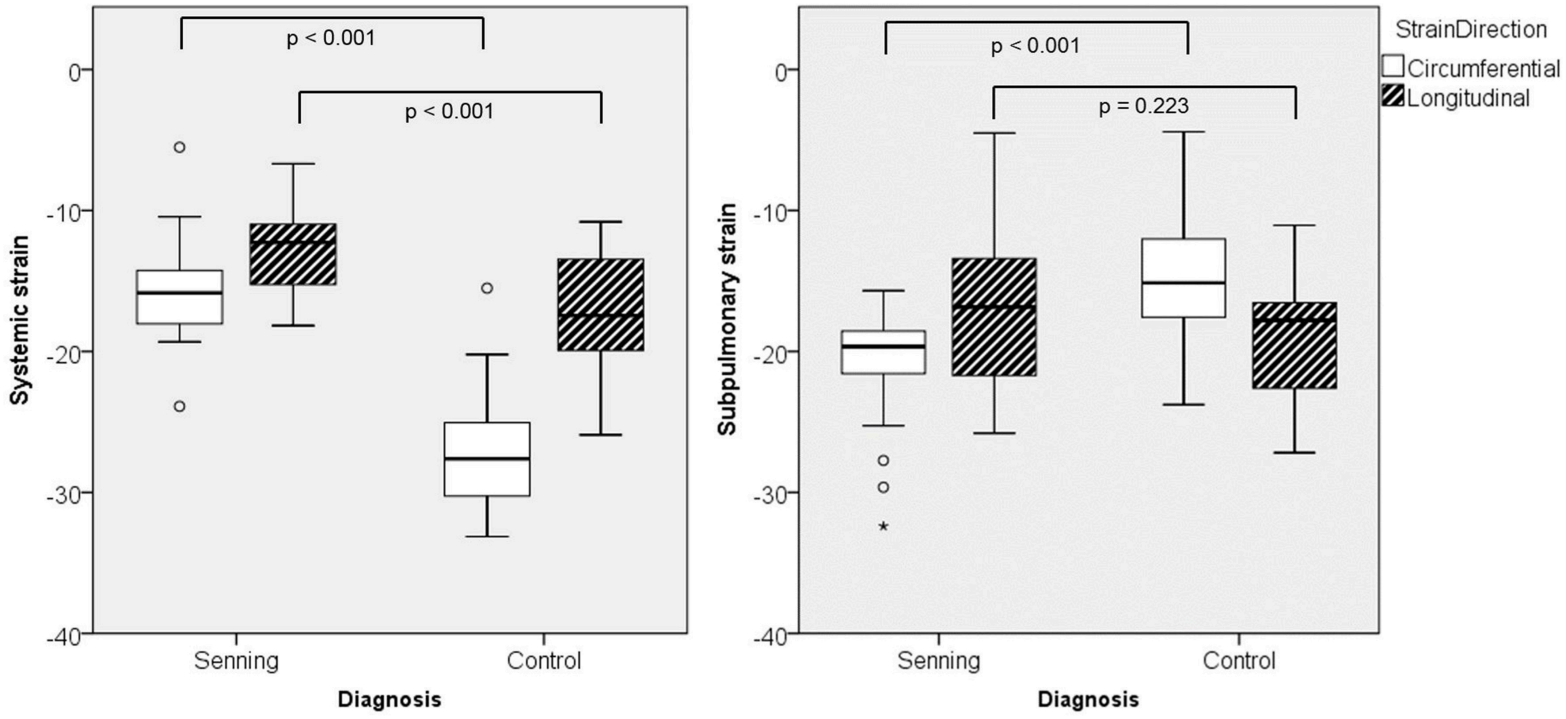
Maximal global strain for systemic and subpulmonary ventricles in Senning patients and controls.

Comparing deformation parameters in relation to the functional position of the ventricle, the systemic RV had lower global longitudinal and circumferential strains compared to the normal systemic LV (*p* < 0.001) (Figure 3; Supplementary Table 2).

Both ventricles in subpulmonary position, i.e., the RV in normal subjects and the LV in Senning patients, showed similar global longitudinal strain, however circumferential strain was higher in the LV than in the RV (*p* < 0.001).

The systemic RV in Senning patients showed significantly reduced twist (−3.1 ± 11 vs. 22.3 ± 7.4°; *p* < 0.001) and reduced torsion (−0.6 ± 1.9 vs. 3.8 ± 1.2 °/cm; *p* < 0.001) compared to the systemic LV in controls.

Considering regional deformation, in Senning patients, strain values were generally higher in the free wall of both ventricles compared to global strain values. In normal subjects, free wall strain values were higher in longitudinal but not in circumferential direction (Table 3).

**Table 3:** Comparison of global vs. free wall strains.

	**Global**	**Free wall**	***p***
**SENNING**			
RV longitudinal strain [%]	−12.9 ± 3.3	−15.3 ± 3.2	**<0.001**
RV circumferential strain [%]	−15.8 ± 3.4	−17.1 ± 3.3	**0.003**
LV longitudinal strain [%]	−17 ± 5.6	−20.4 ± 8.1	**0.001**
LV circumferential strain [%]	−20.7 ± 4.1	−24.3 ± 5.1	**<0.001**
**CONTROLS**			
RV longitudinal strain [%]	−18.9 ± 4.6	−24.7 ± 5.4	**<0.001**
RV circumferential strain [%]	−15.1 ± 5	−15.2 ± 6.7	0.92
LV longitudinal strain [%]	−17.5 ± 4.6	−20.4 ± 4.1	**0.002**
LV circumferential strain [%]	−27.3 ± 4.5	−26.7 ± 4.7	0.148

Values are expressed as mean ± standard deviation. LV, left ventricle; RV, right ventricle. p values in bold type denote statistically significant differences

### Strain and Ventricular Volume

In Senning patients, longitudinal RV strain correlated with RV EF% (*r* = −0.554; *p* < 0.01), as well as with LV EF% (*r* = −0.468; *p* < 0.05). Circumferential strain of the systemic RV correlated with RV EDV (*r* = 0.705; *p* < 0.001), LV EDV (*r* = 0.425; *p* < 0.05), and RV EF% (*r* = −0.706; *p* < 0.001). LV longitudinal or circumferential strains did not correlate with ventricular volumes or EF%.

In the control group, RV longitudinal strain correlated with RV EF% (*r* = −0.592; *p* < 0.01) and with LV EF% (*r* = −0.591; *p* < 0.01).

Comparing subgroups of patients with RV EF% < 40% vs. ≥ 40%, those with lower RV EF% (*n* = 16) had worse RV global (*p* = 0.03) and free wall (*p* = 0.008) longitudinal and worse RV global (*p* = 0.002) and free wall (*p* = 0.008) circumferential strain. LV strain values were not influenced by RV EF%.

RV longitudinal strain correlated with RV circumferential strain in Senning patients (*r* = 0.489; *p* = 0.01), but not in controls.

LV longitudinal strain did not correlate with other strain parameters; LV circumferential strain correlated with RV longitudinal (*r* = 0.753; *p* < 0.001) and circumferential (*r* = 0.615; *p* = 0.005) strains in normal subjects, but not in Senning patients.

### Functional Assessment

Twenty of 29 (69%) Senning patients underwent CPEX within a median interval to CMR of 7 days (range −343 to 346 days). Additionally, the latest CPEX during follow up was considered for further functional assessment and was available in 20 of 29 (69%) patients. Most CPEX were performed as bicycle ergometries using a ramp protocol. Work load [Watts], maximal oxygen consumption [ml/kg/min], and the slope of VE/VCO2 (respiratory equivalent for carbon dioxide) were recorded. Patients were stratified into Weber class (A to D) according to Guazzi et al. (19), analogous to patients with heart failure, pulmonary arterial hypertension, or chronic obstructive pulmonary disease. Due to the retrospective nature of this study, some values were missing in some patients.

At the first CPEX, patients attained a work load of 158 ± 48 Watts and 28.9 ± 5.3 ml/(kg^*^min) peak oxygen consumption, corresponding to 74.9 ± 14.2% predicted. VE/VCO2 was available in 17/29 (59%) of patients, with a mean ± SD of 25.7 ± 3.9. All patients attained Weber class A. VO2max at the time of CMR did not correlate with any ventricular strain. VE/VCO2 correlated weakly with global (*r* = 0.484; *p* = 0.049) and free wall (*r* = 0.509; *p* = 0.037) circumferential RV strain.

At the most recent CPEX, mean performance was 170 ± 56 Watts and 28.4 ± 6.7 ml/(kg^*^min) peak oxygen consumption, corresponding to 72.8 ± 13.7% predicted. All patients except one (Weber class B) were still in Weber class A at the most recent CPEX. VE/VCO2 was measured in 15/20 (52%) of patients at the most recent follow up, with a mean ± SD of 26.7 ± 3.4. No correlations with RV or LV strain were found. Exercise performance was unchanged over a follow-up period of 3.2 ± 1.4 years.

During follow up, patients with an RV EF% < 40% (*n* = 16) were not performing worse than those with RV EF% ≥ 40% (*n* = 13). Performance in Watts, peak oxygen uptake, or percent predicted peak oxygen uptake were not correlated to RV EF%. Long-term exercise performance was not correlated with RV strain values.

One patient moved abroad after the CMR. All others (*n* = 28) were still alive after a follow up time of 53 ± 25 months (range 2–108 months).

### Medication

Senning patients taking ACE inhibitors or angiotensin receptor blockers (*n* = 8) had higher end-diastolic ventricular volumes than their counterparts (RV EDV 131 ± 27 vs. 107 ± 20 ml/m2; *p* = 0.024; LV EDV 86 ± 16 vs. 70 ± 12 ml/m2; *p* = 0.009). Medicated patients also differed from other Senning patients in that they had lower circumferential strains in their systemic RV (global *p* = 0.001; free wall *p* = 0.022). Senning groups did not differ in biventricular EF%, APSE, systemic RV twist or torsion.

### Image Quality

Short axis images were all of sufficient quality for strain analysis. In Senning patients, quality was good in 90% and moderate in 10%. In normal subjects, quality was good in 63% and moderate in 37%.

Long axis image quality was insufficient for strain analysis only in 2 patients after atrial repair. In all other patients, quality was classified as good in 62% and moderate in 31%. In controls, all long axis images could be postprocessed, 90% being of good and 10% of moderate quality. Variations in RR intervals during acquisition or difficulty in tracing the basal septum were the most important factors influencing image quality.

Inadequate quality images were excluded from analysis. Therefore, images were available for analysis of circumferential strain in 29 and for longitudinal strain in 27 patients. We decided not to study radial strain because of its low reproducibility described previously (20).

### Reproducibility

Coefficients of variation and intraclass correlation coefficients demonstrated good reproducibility for intra- and interobserver measurements (Tables 4, 5). Variability was largest for longitudinal measurements in both ventricles.

**Table 4:** Intraobserver variability.

	**Mean value (%)**	**Mean difference (%)**	**SD of differences (%)**	**Limits of agreement (%)**	**CV (%)**	**ICC**
RV ε circ	−17.2	0.67	3.2	−5.7; 7.0	18.8	0.892
LV ε circ	−25.1	−0.94	2.7	−6.3; 4.4	10.9	0.946
RV ε long	−19.1	−0.91	1.5	−3.8; 2.0	7.7	0.983
LV ε long	−16.8	−3.32	3.5	−10.2; 3.6	21.1	0.885

*Limits of agreement encompass the 95% confidence interval of differences between measurements*.

*CV, coefficient of variation = SD of differences between 2 measurements, divided by mean of 2 measurements. ICC, intraclass correlation coefficient; LV ε circ, left ventricular circumferential strain; LV ε long, left ventricular longitudinal strain; RV ε circ, right ventricular circumferential strain; RV ε long, right ventricular longitudinal strain; SD, standard deviation*.

**Table 5:** Interobserver variability.

	**Mean value (%)**	**Mean difference (%)**	**SD of differences (%)**	**Limits of agreement (%)**	**CV (%)**	**ICC**
RV ε circ	−16.1	−1.49	2.8	−7.0; 4.0	17.5	0.908
LV ε circ	−24.6	0.01	2.7	−5.3; 5.3	10.9	0.947
RV ε long	−17.6	2.01	3.9	−5.6; 9.7	22.2	0.862
LV ε long	−15.9	−1.60	4.2	−9.8; 6.6	26.3	0.742

*Limits of agreement encompass the 95% confidence interval of differences between measurements*.

*CV, coefficient of variation; ICC, intraclass correlation coefficient; LV ε circ, left ventricular circumferential strain; LV ε long, left ventricular longitudinal strain; RV ε circ, right ventricular circumferential strain; RV ε long, right ventricular longitudinal strain; SD, standard deviation*.

## Discussion

This study used MRFT for measurement of global and regional strain of both ventricles in patients after atrial switch repair for TGA compared to healthy controls. Our results give new insights into myocardial mechanics and demonstrate that the subaortic RV presents decreased longitudinal deformation compared to a normal subpulmonary RV and to a normal subaortic LV, but unchanged circumferential deformation. As longitudinal shortening represents the main contraction mechanism of the RV, this results in a decreased RF EF%. The inability to increase circumferential strain is a further finding indicating that the myocardium of the subaortic RV is unable to adequately adapt to a chronic pressure overload. Furthermore, the RV cannot mimic the rotational mechanism of an LV in systemic position. The LV in subpulmonary position shows a decrease in circumferential strain, while maintaining longitudinal deformation; this reflects the remodeling in a pressure unloaded LV.

Dysfunction of the subaortic RV and eventually right heart failure are a major determinant of outcome in patients after the atrial switch operation for TGA (3). Impairment of global contractility measured as EF% is considered a late finding; therefore, more sensitive methods are needed for timely detection of RV dysfunction. RV myocardial strain by speckle tracking echocardiography has been shown to correlate with other biomarkers, functional class (21), and outcome (10, 11). In Senning patients and others with a systemic and dilated RV, MRFT represents a useful alternative modality to speckle tracking, as it overcomes the well-known limitations of echocardiography for imaging the entire dilated RV. Since CMR images are usually taken in a short axis covering both ventricles, a standard plane for measuring ventricular volumes, MRFT can obtain not only global longitudinal strain, but also circumferential strain (22). MRFT has clear advantages compared to other CMR techniques for evaluation of myocardial deformation, such as myocardial tagging or displacement encoding with stimulated echoes (DENSE), which are sophisticated sequences, potentially challenging to acquire and with cumbersome post-processing (23). In fact, MRFT can be easily applied on standard cine SSFP images, if temporal and spatial resolutions are sufficient (20). MRFT has been validated against other deformation sequences, and excellent agreement between MRFT and tagged harmonic phase analysis has been reported (24). Our data are very similar to normal endocardial MRFT reference values (25) and to strain values reported recently in a larger cohort of adults after atrial repair (13).

The decreased longitudinal strain which we have found in the subaortic RV is in agreement with data previously reported by tissue Doppler echocardiography (26, 27). We have observed that circumferential strain in the subaortic RV is not increased. Both findings suggest that the subaortic RV in Senning patients not only loses some degree of longitudinal deformation, which is the main force of the RV, but also can not compensate chronic pressure overload by increasing the activity of any circumferential fibers, which are scarce in the RV. In an animal model with pulmonary artery banding, by using diffusion tensor imaging, Nielsen et al. demonstrated that the RV is structurally unable to sustain a permanent increase in afterload (28). Our observation that in the unloaded LV, circumferential strain is higher than in the normal RV similarly reflects the myocardial fiber structure of the ventricles, with a larger layer of well-developed circumferential fibers in the LV. Secondly, CMR studies have described the presence of regional and diffuse RV myocardial fibrosis in a considerable number of patients after the atrial switch operation (29, 30), albeit no correlation has been found with peak oxygen uptake during exercise. From a mechanistic point of view, it seems self-evident that fibrotic myocardium may deform less. Thirdly, additional factors other than solely intrinsic myofiber mechanics may influence deformation. Most recently by using three-dimensional techniques, Morcos et al. comprehensively assessed global and regional function of the systemic RV in atrial switch and in congenitally corrected TGA patients. They found that global and regional myocardial function was more abnormal in the atrial switch patients than in the congenitally corrected TGA group (31). We have previously described an abnormal “atrial” function with a decreased reservoir and pump component in Senning patients and an increased conduit component of atrial dynamics, which corresponds to a loss of atrial pulsatility (32). The results of both studies suggest a relationship between abnormal filling and abnormal ventricular mechanics of the systemic RV. The mechanisms of atrioventricular coupling in this patient population deserve further investigation.

We have observed a positive correlation between RV strain and EF% with LV EF%. This finding is in line with the well-known interventricular interaction. Interventricular interaction in patients after atrial switch or with congenitally corrected TGA has also been demonstrated by echocardiographic speckle tracking analysis (10).

A very interesting finding of this study is that in Senning patients strain values of the free walls were higher than global strain values in both ventricles. We hypothesize that the abnormal D-shape of the septum with a pressure overloaded RV may negatively affect septal myocardial mechanics and lead to decreased septal strain values; this obviously has a direct influence on values of global deformation, but not on deformation values of the free walls. Therefore, we suggest that in Senning patients, measurement of free ventricular wall strain may better reflect the myocardial deformation and performance of both ventricles, rather than global strain.

Many Senning patients presented with a reduced exercise capacity, which remained stable over the follow up time of 3.5 years. The lack of significant correlations between the CMR parameters and CPEX results may be explained by the fact that CMR parameters were acquired at rest rather than during exercise. In fact, it has been shown that atrially switched patients cannot increase stroke volume during stress in contrast to patients with congenitally corrected transposition (33). The reason for this is most probably the abnormal “atrial” function described above (32) causing an abnormal preloading of the ventricles, which may be even better unmasked during exercise. As the atrial switch patient cohort ages, correlations between CMR parameters and adverse events in the natural course should ideally be evaluated prospectively. This cross-sectional study provides a basis toward this.

We found acceptable inter- and intraobserver reproducibility of MRFT measurements, for both longitudinal and circumferential strain. The slightly larger variability for longitudinal measurements is similar to that described by other authors and can be explained by the difficulties in correctly tracking the most basal myocardial segments in the longitudinal direction throughout the cardiac cycle, due to the strong in-plane motion of the AV valves (20).

## Limitations

This is a monocentric, retrospective study with a limited number of patients; thus, our results must be considered preliminary. Due to the retrospective nature of the study, we could not analyze the effect of fibrosis on myocardial deformation, since late gadolinium enhancement data were not available in all patients, and T1 mapping was not performed at the time of data acquisition.

Our data may have been influenced by a referral bias, since patients with poor function and arrhythmias or pacemakers, as well as patients with very good RV function on echocardiography may have not been referred for CMR. However, this aspect was investigated by Tutarel et al., who did not report any significant difference in strain values between the overall atrial switch group and a subgroup of patients in optimal clinical condition (13).

## Conclusion

Myocardial deformation of the subaortic RV after atrial switch operation for TGA as measured by MRFT is abnormal. In Senning patients, the subaortic RV has decreased longitudinal and unchanged circumferential deformation. These data suggest that the subaortic RV is not able to adapt to chronic pressure overload and to remodel to mimic a LV. Myofiber architecture, intrinsic myofiber function, and other mechanisms, including myocardial fibrosis as well as an abnormal preload may be potential explanations.

## Ethics Statement

This study was carried out in accordance with the recommendations of the ethics committee of the canton of Zurich, Switzerland with written informed consent from all subjects. All subjects gave written informed consent in accordance with the Declaration of Helsinki. The protocol was approved by the ethics committee of the canton of Zurich, Switzerland (KEK-ZH-Nr. 2015–0120).

## Author Contributions

BB and EV contributed to conception and design of the study and made measurements. BB acquired ethics approval. BB, CK, JG, AO, and EV acquired data. BB performed the statistical analysis and drafted the manuscript. CK, JG, FF, AO, and EV revised it critically for important intellectual content. All authors provided approval for publication of the manuscript.

### Conflict of Interest Statement

The authors declare that the research was conducted in the absence of any commercial or financial relationships that could be construed as a potential conflict of interest.
