# Supplementary material for: Right and Left Ventricular Strain Patterns After the Atrial Switch Operation for D-Transposition of the Great Arteries—A Magnetic Resonance Feature Tracking Study
Source: Front Cardiovasc Med. 2019 Apr 9;6:39. doi: 10.3389/fcvm.2019.00039 (PMC6465947; doi:10.3389/fcvm.2019.00039)
Supplement: Supplementary file 1 [file Table_1.DOC]

**Supplementary table 1:** Global and free wall ventricular strains comparing the ventricles by their anatomy (RV in systemic position with RV in subpulmonary position; LV in systemic position with LV in subpulmonary position)

|  |  | Senning | Control | p |
| --- | --- | --- | --- | --- |
| RV longitudinal strain [%] | | | | |
|  | global | -12.9 +/- 3.3 | -18.9 +/- 4.6 | **< 0.001** |
|  | *free wall* | *-15.3 +/- 3.2* | *-24.7 +/- 5.4* | ***< 0.001*** |
| RV circumferential strain [%] | | | | |
|  | global | -15.8 +/- 3.4 | -15.1 +/- 5 | 0.567 |
|  | *free wall* | *-17.1 +/- 3.3* | *-15.2 +/- 6.7* | *0.267* |
| LV longitudinal strain [%] | | | | |
|  | global | -17 +/- 5.6 | -17.5 +/- 4.6 | 0.711 |
|  | *free wall* | *-20.4 +/- 8.1* | *-20.4 +/- 4.1* | *0.415* |
| LV circumferential strain [%] | | | | |
|  | global | -20.7 +/- 4.1 | -27.3 +/- 4.5 | **< 0.001** |
|  | *free wall* | *-24.3 +/- 5.1* | *-26.7 +/- 4.7* | *0.056* |

Values are expressed as mean +/- standard deviation.

LV – left ventricle

RV – right ventricle
