# Supplementary material for: Right and Left Ventricular Strain Patterns After the Atrial Switch Operation for D-Transposition of the Great Arteries—A Magnetic Resonance Feature Tracking Study
Source: Front Cardiovasc Med. 2019 Apr 9;6:39. doi: 10.3389/fcvm.2019.00039 (PMC6465947; doi:10.3389/fcvm.2019.00039)
Supplement: Supplementary file 2 [file Table_2.DOC]

**Supplementary table 2:** Global and free wall ventricular strains comparing the ventricles by functional position (systemic and subpulmonary)

|  |  | Senning: RV | Control: LV | p |
| --- | --- | --- | --- | --- |
| Systemic longitudinal strain [%] | | | | |
|  | global | -12.9 +/- 3.3 | -17.5 +/- 4.6 | **< 0.001** |
|  | *free wall* | *-15.3 +/- 3.2* | *-20.4 +/- 4.1* | ***< 0.001*** |
| Systemic circumferential strain [%] | | | | |
|  | global | -15.8 +/- 3.4 | -27.3 +/- 4.5 | **< 0.001** |
|  | *free wall* | *-17.1 +/- 3.3* | *-26.7 +/- 4.7* | ***< 0.001*** |
|  | | | | |
|  | | Senning: LV | Control: RV | p |
| Subpulmonary longitudinal strain [%] | | | | |
|  | global | 17 +/- 5.6 | -18.9 +/- 4.6 | 0.223 |
|  | *free wall* | *-20.4 +/- 8.1* | *-24.7 +/- 5.4* | *0.084* |
| Subpulmonary circumferential strain [%] | | | | |
|  | global | -20.7 +/- 4.1 | -15.1 +/- 5 | **< 0.001** |
|  | *free wall* | *-24.3 +/- 5.1* | *-15.2 +/- 6.7* | ***< 0.001*** |

Values are expressed as mean +/- standard deviation.

LV – left ventricle

RV – right ventricle
